# Histone H2A (H2A.X and H2A.Z) Variants in Molluscs: Molecular Characterization and Potential Implications For Chromatin Dynamics

**DOI:** 10.1371/journal.pone.0030006

**Published:** 2012-01-11

**Authors:** Rodrigo González-Romero, Ciro Rivera-Casas, Lindsay J. Frehlick, Josefina Méndez, Juan Ausió, José M. Eirín-López

**Affiliations:** 1 Departamento de Biología Celular y Molecular, Universidade da Coruña, A Coruña, Spain; 2 Department of Biochemistry and Microbiology, University of Victoria, Victoria, Canada; Florida State University, United States of America

## Abstract

Histone variants are used by the cell to build specialized nucleosomes, replacing canonical histones and generating functionally specialized chromatin domains. Among many other processes, the specialization imparted by histone H2A (H2A.X and H2A.Z) variants to the nucleosome core particle constitutes the earliest response to DNA damage in the cell. Consequently, chromatin-based genotoxicity tests have been developed in those cases where enough information pertaining chromatin structure and dynamics is available (i.e., human and mouse). However, detailed chromatin knowledge is almost absent in most organisms, specially protostome animals. Molluscs (which represent sentinel organisms for the study of pollution) are not an exception to this lack of knowledge. In the present work we first identified the existence of functionally differentiated histone H2A.X and H2A.Z variants in the mussel *Mytilus galloprovincialis* (MgH2A.X and MgH2A.Z), a marine organism widely used in biomonitoring programs. Our results support the functional specialization of these variants based on: a) their active expression in different tissues, as revealed by the isolation of native MgH2A.X and MgH2A.Z proteins in gonad and hepatopancreas; b) the evolutionary conservation of different residues encompassing functional relevance; and c) their ability to confer specialization to nucleosomes, as revealed by nucleosome reconstitution experiments using recombinant MgH2A.X and MgH2A.Z histones. Given the seminal role of these variants in maintaining genomic integrity and regulating gene expression, their preliminary characterization opens up new potential applications for the future development of chromatin-based genotoxicity tests in pollution biomonitoring programs.

## Introduction

In eukaryotes, the availability of the genetic information stored in the DNA is modulated by the interactions of this molecule with a broad spectrum of nuclear proteins, among which, histones represent key players in the regulation of DNA metabolism [1]. Histones are a group of small basic proteins responsible for the packing and compactation of DNA in the cell nucleus, constituting the chromatin fiber. These proteins can be grouped into two major categories: core histones (H2A, H2B, H3 and H4) and linker histones (H1 family). The interaction between core histones leads to the formation of an octameric core structure around which 146 bp of DNA are wrapped in approximately one and three quarter left handed superhelical turns, giving rise to the nucleosome core particle (NCP), a highly dynamic nucleoprotein complex which constitutes the fundamental packaging subunit of chromatin [2]. In contrast, linker histones bind to regions connecting adjacent nucleosomes in the chromatin fiber, mediating chromatin compaction through the assembly of higher order chromatin structures [3]. However, the role played by these proteins goes far beyond these structural considerations. They additionally have critical functions for chromatin metabolism (including DNA transcription, replication, recombination and repair, among other processes) by regulating the access of different cellular components to DNA [4]. The different ways in which histones can affect chromatin metabolism constitute what has been defined as the ‘histone language’ which is based on a ‘histone code’ [5], [6]. Such code results from the combination of histone post-translational modifications (PTMs) [7] and the specialization imparted to chromatin by the exchange of canonical histones by specialized histone variants [8].

Each histone family encompasses a set of minoritary variants, in addition to the main group of canonical proteins, all of them being amenable for PTMs [9]. Among core histones, the H2A family is of great interest due to the high diversity of specialized variants it displays, including proteins involved in critical cellular processes [6]. Indeed, two H2A variants stand out regarding their functional relevance: on one hand, the histone H2A.X is involved in apoptosis, meiosis and replication through its role in the maintenance of genome integrity [6], [10]. Upon DNA Double-Strand Breaks (DSBs), H2A.X histones of extensive regions flanking a damaged site become reversibly phosphorylated at their C-terminal SQEY motif (γ-H2A.X) creating the so-called ‘H2A.X foci’. This mechanism promotes the dynamic remodeling of chromatin, constituting the primary signal activating the mechanism of DNA DSB repair within the cell nucleus [10], [11]. On the other hand, histone H2A.Z plays critical roles in gene regulation as well as in the establishment of chromatin boundaries [12], [13]. Furthermore, different reports have directly or indirectly suggested the participation of H2A.Z in the maintenance of genome integrity. For instance, the exchange of γ-H2A.X with H2A.Z seems to facilitate the recruitment of DNA repair factors and checkpoint factors [14], [15], [16]. The interest in the study of this variant is thus reliant on its relevance for cell viability as well as on the controversy raised by its apparently dual function in regulating gene activation/repression [17], [18].

The rekindling of the interest in chromatin research during the last 20 years has led to a careful characterization of chromatin structure and dynamics in a wide range of model systems, most notably mammals. However, detailed studies on this matter are very limited in almost any other group [19], most notably in the case of protostome animals. Molluscs are of special interest for the study of chromatin within this latter group due to two main reasons: first, histone genes are subject to an intense diversification process within this taxonomic group [20], [21], [22]; and second, bivalve molluscs are sentinel organisms widely used in the biomonitoring of pollution in the marine environment, encompassing outstanding economic relevance for the aquaculture industry [23]. Within this frame, the close connection between the genotoxic effect of different pollutants in the marine environment (*i.e.*, marine biotoxins, oil spills) and the role played by H2A.X and H2A.Z variants in the maintenance of genome integrity opens up a very interesting research pipeline with dual benefits: first, to shed light on the mechanisms underlying chromatin metabolism and dynamics in molluscs; and second, to develop quick and efficient chromatin-based genotoxicity tests in pollution biomonitoring programs [23]. In the present work we identify for the first time the presence of functionally differentiated histone H2A.X and H2A.Z variants in the mussel *Mytilus galloprovincialis*, a marine mollusc widely used in biomonitoring programs. Our results suggest that these variants are functionally specialized, based on their active expression, their evolutionary conservation as well as the specialization they impart to nucleosomes. The implications of these results for the development of chromatin-based genotoxicity tests are discussed here.

## Results and Discussion

### Histone H2A.X and H2A.Z gene variants are present in the genome of the mussel *Mytilus galloprovincialis*

**Figure 1:**
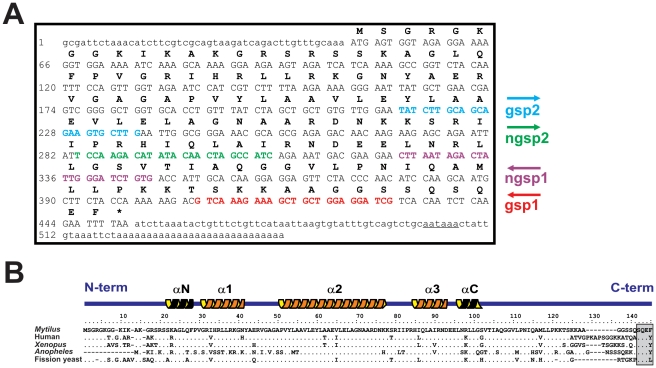
Nucleotide and protein sequences determined for the histone H2A.X variant in *M. galloprovincialis*. A) Nucleotide and corresponding protein sequences encoded by the H2A.X cDNA isolated by RACE (GenBank accession number HQ24264). The 5′ and 3′ UTR regions are indicated in lowercase, the coding region in capitals, the protein sequence in capitals and boldface, and the polyadenylation site is underlined. Internal primers designed for RT-PCR and RACE experiments (boldface colored DNA sequences) are indicated in the right margin of the sequence. B) Protein sequence alignment of different H2A.X proteins (GenBank accession numbers: Human, NM_002105; *Xenopus laevis*, NM_001092630; *Anopheles gambiae*, XM_320674; *Schizosaccharomyces pombe*, NM_001023170). The amino (N) and carboxy (C) terminal ends of the H2A.X protein, as well as the secondary structure of the histone fold motif, are represented above the alignment. The archetypal SQEY motif involved in the formation of the H2A.X *foci* is indicated in grey background.

**Figure 2:**
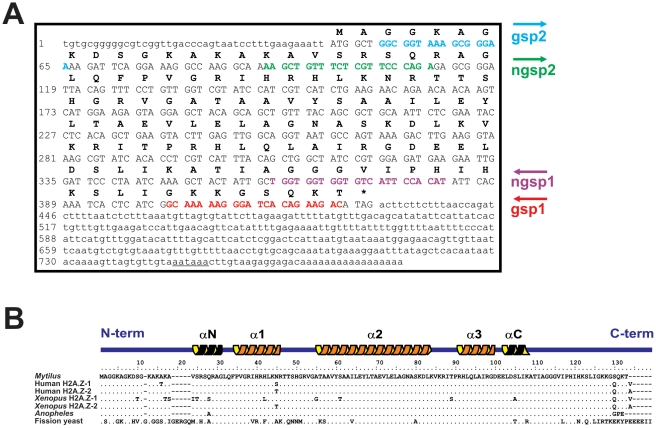
Nucleotide and protein sequences of the histone H2A.Z variant in *M. galloprovincialis*. A) Nucleotide and protein sequences encoded by the H2A.Z cDNA isolated by RACE (GenBank accession number HQ242649). The 5′ and 3′ UTR regions, coding region, protein sequence, polyadenylation site and internal primers are indicated as in Fig. 1. B) Protein sequence alignment of different H2A.Z proteins (GenBank accession numbers: Human H2A.Z-1, NM_002106; Human H2A.Z-2, NM_012412; *Xenopus laevis* H2A.Z-1, NM_001086059; *Xenopus laevis* H2A.Z-2, NM_001092643; *Anopheles gambiae*, XM_310818; *Schizosaccharomyces pombe*, NM_001021524). The amino (N) and carboxy (C) terminal ends of the H2A.Z protein, as well as the secondary structure of the histone fold motif, are represented above the alignment.

In the present work, the genes encoding histone H2A.X and H2A.Z variants have been identified for the first time in a bivalve mollusc (the mussel *M. galloprovincialis*). The complete cDNA sequences have been deposited in GenBank under the accession numbers HQ242648 (MgH2A.X) and HQ242649 (MgH2A.Z). Complete cDNA sequences were obtained from polyadenylated mRNAs extracted from male gonad using RT-PCR and RACE experiments. The sequence corresponding to histone H2A.X (MgH2A.X, Fig. 1A) consisted of 548 bp, containing a 5′ untranslated region (UTR) of 47 bp, a 3′ UTR of 96 bp, and an open reading frame (ORF) of 405 bp encoding a H2A.X protein of 134 residues. In the case of H2A.Z (MgH2A.Z, Fig. 2A) the cDNA sequence consisted of a ORF of 384 bp encoding a MgH2A.Z protein of 127 residues, flanked by 5′ and 3′ UTR regions of 43 bp and 359 bp, respectively. Both sequences exhibited canonical polyadenylation signal sites (AATAAA) and a polyadenylation tail at 3′ UTR regions.

The amino acid sequence obtained for MgH2A.X is characteristic by showing a SQEF motif located at the C-terminal region of the protein (highlighted in grey background in Fig. 1B), displaying a high degree of similarity to the archetypal SQEY motif specific to most vertebrate H2A.X proteins. Given that this motif represents a consensus sequence for kinases (involved in the phosphorylation of the serine residue) during the early response to DNA double-strand breaks [10], [24], its presence in MgH2A.X could be potentially linked to a similar role in this group of molluscs. Indeed, comparisons among primary H2A.X protein structures across different organisms revealed a high degree of conservation of the protein region containing this motif (Fig. 1B), lending further support to the presence of intense selective constraints preserving the function on this variant. A high degree of protein conservation is even more evident in the case of H2A.Z proteins (Fig. 2B), emphasizing the functional relevance of these variants across the evolutionary scale. However, contrary to the case of H2A.Z from chordates, MgH2A.Z lacks the characteristic triresidue signature, displaying only the two first residues (alanine, asparagine) at positions 16 and 45 in the alignment shown Fig. 2B [18]. The primary structure displayed by MgH2A.Z is similar to that found in different protostomes and consistent with the stepwise mutation model for the origin of vertebrate H2A.Z-1 and H2A.Z-2 proteins [17], [18].

**Figure 3:**
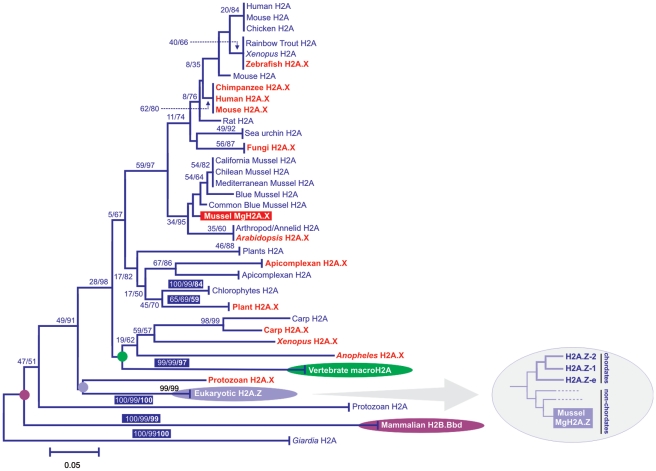
Phylogenetic relationships among histone H2A proteins from different eukaryotes (see [**18**]), indicating the evolutionary position of MgH2A.X and MgH2A.Z histones (highlighted in red and light-blue backgrounds, respectively). The different H2A types are indicated in the topology, including canonical H2A proteins (blue), and the variants H2A.X (red), macroH2A (green), the H2A.Z fraction (light-blue) and H2A.Bbd (purple). Circles indicate the monophyletic origin for the corresponding group of variants. MgH2A.X, similarly to H2A.X proteins from other organisms, appears interspersed with canonical H2A types, showing a close relationship with canonical H2A histones from molluscs. MgH2A.Z is, on the other hand, clustered within the monophyletic group encompassing eukaryotic H2A.Z proteins, closely related to H2A.Z proteins from non-chordate organisms (see subtree in the right handside of the topology). Numbers for interior nodes indicate BS/CP confidence values. Numbers in colored boxes and in boldface account for the bootstrap values obtained in the reconstruction of the maximum parsimony trees using all the informative positions in the alignment. Confidence values were based on 1000 replications and are only shown if at least one of the values is >50%.

The phylogeny shown in Fig. 3 summarizes the evolutionary relationships reconstructed for H2A proteins in several groups of eukaryotes (see [18] for a complete list of the sequences used and their accession numbers), including histone H2A.Bbd [25], [26], the group comprising H2A.Z variants [22] and macroH2A [27], [28]. While the aforementioned H2A variants display a common feature (they all have monophyletic origins), histone H2A.X is peculiar by showing a recurrent process of differentiation across evolution, implying that it has had multiple evolutionary origins as previously reported [10], [29]. When examining the position occupied by *Mytilus* H2A variants in the tree topology it is evident that MgH2A.X appears interspersed with canonical H2A types (similarly to H2A.X proteins from other organisms), showing a close relationship with canonical H2A histones from molluscs. On the other hand, MgH2A.Z clusters within the monophyletic group encompassing eukaryotic H2A.Z proteins, closely related to H2A.Z proteins from non-chordate organisms (see subtree in the right handside of the topology). Overall, our results suggest that genes encoding MgH2A.X and MgH2A.Z are functionally transcribed in the genome of the mussel *Mytilus*, encoding histone variants highly similar to those of deuterostomes probably as a consequence of the presence of strong functional constraints leading to their evolutionary conservation.

### MgH2A.X and MgH2A.Z are actively expressed in different tissues

**Figure 4:**
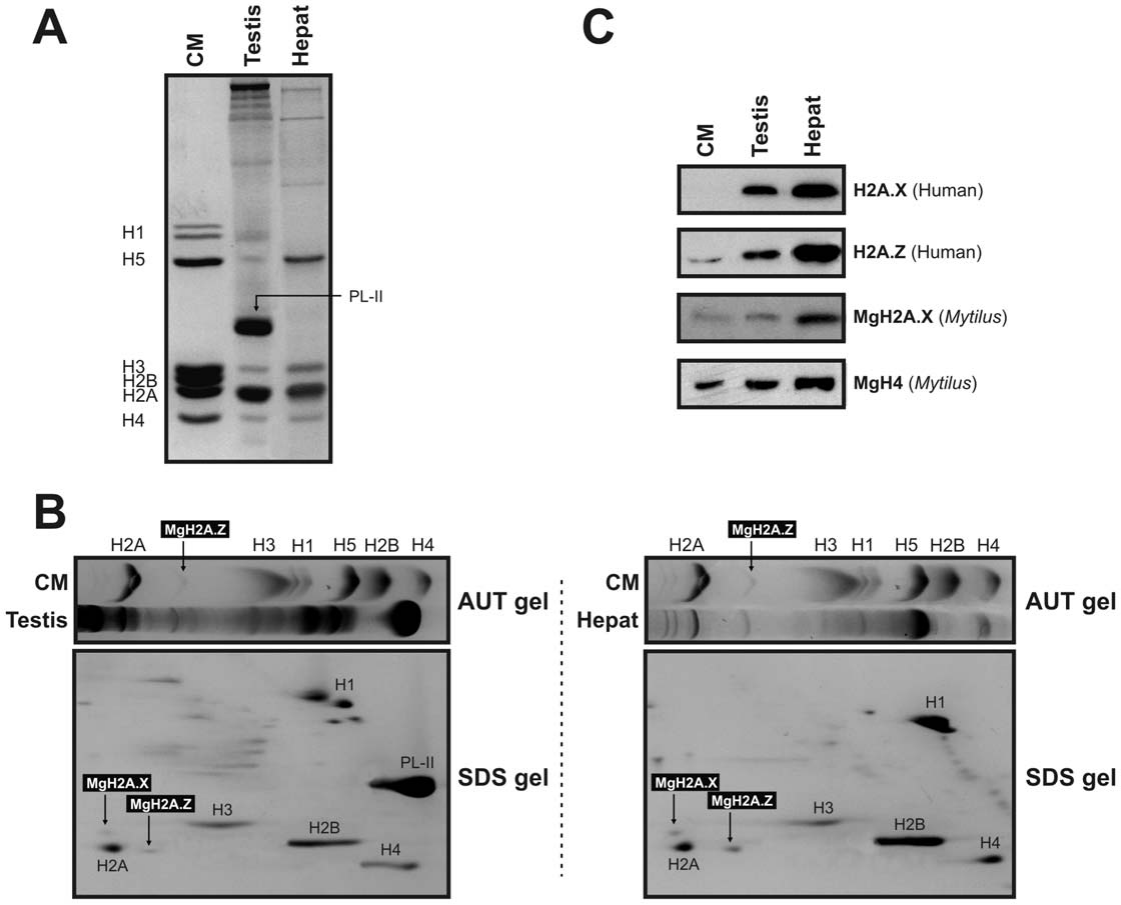
Electrophoretic characterization of *Mytilus* histones and Western blot analyses of MgH2A.X and MgH2A.Z variants in different tissues. A) SDS-PAGE separation of native histones extracted from gonad and hepatopancreas. B) Two-dimensional PAGE analysis revealing the presence of native MgH2A.X and MgH2A.Z proteins in gonad and hepatopancreas extracts. C) Western blot experiments on gonad and hepatopancreas using human-specific anti-H2A.X and anti-H2A.Z antibodies, as well as a specific antibody raised against H2A.X from *Mytilus*, further corroborating the presence of MgH2A.X and MgH2A.Z in these tissues. CM, chicken erythrocyte histones used as marker. MgH4 (*Mytilus*), loading control for Western experiments.

Given the extensive reorganization experienced by chromatin in the germinal cell line (involving DNA breaks/repair during meiosis), as well as the potential bioaccumulation of genotoxic pollutants in the hepatopancreas (digestive gland) of mussels, both tissues were chosen as good candidates for studying the expression and potential role of MgH2A.X and MgH2A.Z in the maintenance of genome integrity. With this aim, *Mytilus* histone proteins were extracted and analyzed using denaturing conditions in sodium dodecyl sulphate (SDS) and acid-urea-triton (AUT) gels. As depicted by Fig. 4A, the gonad of *Mytilus* contains histones with an electrophoretic mobility which is similar to that of somatic histones, as well as protamine-like proteins such as PL-II. These latter proteins replace most of the histone complement during spermiogenesis but coexist with approximately 20% of the somatic complement of histones in the mature sperm [30], [31]. Interestingly, SDS gels revealed the presence of mussel H1 proteins showing similarities (in size and mobility) with the highly specialized histone H5 variant from chicken, supporting the replication-independent nature of histone H1 in molluscs [22], [32]. In addition, the identity of *Mytilus* histones was further confirmed by two-dimensional PAGE analyses using AUT for the first dimension and SDS for the second dimension (Fig. 4B). Although the occurrence of MgH2A.Z both in gonad and hepatopancreas is clearly observed in the AUT gels, the high similarity between MgH2A.X and canonical H2A proteins hampers the independent identity of both histone types in a single band, its presence becoming only evident after the electrophoretic separation in the second dimension.

In addition to revealing the presence of native MgH2A.X and MgH2A.Z proteins in *Mytilus*, a further step into the detection and characterization of these variants was undertaken by developing specific antibodies. In an initial experiment, two independent commercial human-specific anti-H2A.X (ABM) and anti-H2A.Z antibodies (ABCAM) were preliminary used in Western blot experiments on gonad and hepatopancreas extracts from *Mytilus*. The results obtained (Fig. 4C) revealed the presence of positive signals in both tissues, demonstrating the ability of these antibodies in detecting the presence of MgH2A.X and MgH2A.Z proteins. However, with the aim of developing a molecular routine specific for the mussel *Mytilus*, two primary polyclonal antibodies were raised against MgH2A.X and MgH2A.Z in order to detect these proteins *in vivo*. In this latter case, only the Western blot experiments using the anti-MgH2A.X antibody were successful (Fig. 4C), whereas the anti-H2A.Z antibody failed to detect MgH2A.Z. Our results indicate that both variants are not only transcribed but also translated in gonad and hepatopancreas from *Mytilus*. Furthermore, it looks that MgH2A.X and MgH2A.Z are expressed at higher levels in this latter tissue, opening a new research direction into their differential expression patterns. Given that recent reports have pointed out that the functional relevance of different histone variants could be achieved either through transcribed (mRNAs) or translated (proteins) products [33], the experimental evidence supporting the translation of MgH2A.X and MgH2A.Z into proteins represents a critical result in order to fully understand their role in chromatin dynamics.

### MgH2A.X and MgH2A.Z-containing nucleosomes organize DNA with slightly different compaction levels

Histone variants impart specialized physicochemical features to the nucleosome core particle by dynamically replacing canonical histones, resulting in the remodeling of local chromatin segments associated with specific roles both at structural and functional levels [6]. Accordingly, we have studied the effect of the incorporation of MgH2A.X an MgH2A.Z into the nucleosome core particle of the mussel *Mytilus*. To this end, *Mytilus* native canonical core histones and recombinant MgH2A.X an MgH2A.Z proteins were used to reconstitute histone octamers by mixing stoichiometric amounts of *Mytilus* H2B, H3, and H4 histones with either: (a) native canonical H2A from *Mytilus*, (b) recombinant MgH2A.X, and (c) recombinant MgH2A.Z. Octamers were reconstituted onto a 193 bp DNA template obtained from the promoter region of the heat shock gene hsp90-1 from *M. galloprovincialis*, which represents an environmentally responsive gene [34]. Given the involvement of hsp90-1 in the maintenance of cellular homeostasis [35], [36], [37], [38], its choice in the present work bears critical relevance for future studies of chromatin dynamics associated with environmental genotoxic stress. The suitability of this DNA fragment was further assessed by high nucleosome occupancy scores (as revealed by computational analyses using the NuPoP software, see Materials and Methods), including a p53 binding site encompassing potential applications for future genotoxicity tests in aquaculture industry as well as in cancer research [39], [40], [41], [42].

**Figure 5:**
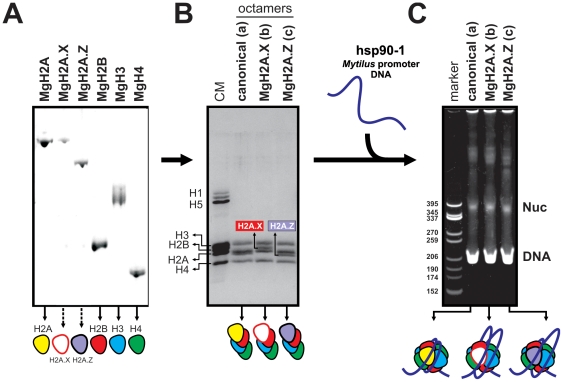
Reconstitution of canonical, MgH2A.X-, and MgH2A.Z-containing nucleosomes. A) Native core histones and recombinant histone variants (MgH2A.X and MgH2A.Z) used in reconstitution experiments. B) Purified histone octamers including canonical H2A, MgH2A.X, and MgH2A.Z histones. CM, chicken erythrocyte histones used as marker. C) Analysis of the nucleosome core particles reconstituted in native PAGE gels. DNA, free DNA; nuc, nucleosome core particles; marker, *Cfo*I digested pBR 322 plasmid DNA used as a marker.

Reconstitution experiments were carried out starting with the purification of the different histone proteins (Fig. 5A) and their subsequent assembly into the three different types (a, b, and c) of nucleosome octamers (Fig. 5B). Complete nucleosomes were successfully reconstituted onto the 193 bp DNA template (nucleosome positioning sequence) from *M. galloprovincialis* (Fig. 5C). The reconstituted particles exhibited similar electrophoretic behavior on native PAGE experiments, although nucleosome core particles containing MgH2A.Z exhibited a slightly higher electrophoretic mobility when compared with particles consisting only of native core histones. This behavior may be indicative of a slightly more compact organization of the MgH2A.Z-nucleosomes, mirroring differences in charge between canonical H2A and MgH2A.Z proteins [43]. On the other hand, the high similarity in mobility between canonical nucleosomes and MgH2A.X-nucleosomes is consistent with the high degree of homology between canonical H2A and MgH2A.X proteins, whose differences are essentially circumscribed to the C-terminal SQEF polypeptide potentially involved in the response to double strand DNA breaks [10].

The present work constitutes the first report describing the presence of histone H2A variants in the chromatin of a mollusc species. Our results demonstrate that both H2A.X and H2A.Z genes are transcribed and translated into proteins in the mussel *M. galloprovincialis*, indicating that the evolutionary differentiation of these had already been achieved in this group of protostomes. Furthermore, the specificity in the functional role of these proteins seems to be supported by the evolutionary conservation of different molecular features, as well as by their ability to confer specialization to nucleosomes (most notably in the case of MgH2A.Z). These results have critical relevance for the characterization of chromatin metabolism and dynamics in protostomes. Furthermore, the characterization of two histone variants (H2A.X and H2A.Z) closely related with the molecular response to genetic damage in bivalve molluscs constitutes a very innovative approach for the future development of molecular assays, using these variants as biomarkers in chromatin-based genotoxicity tests. This latter objective will harbor potential additional applications in conservation genetics, for the optimization of mussel harvesting techniques as well as for the improvement of the quality controls (safety of consumer's health).

## Materials and Methods

### Ethics statement

No specific permits were required for the described field studies. Mussel specimens were collected in the Ría de Arousa (NW Spain, Atlantic coast), in a location which is not privately-owned or protected in any way. The present field studies did not involve endangered or protected species.

### Isolation of H2A.X and H2A.Z histone genes

Total RNA was extracted from male gonad of the mussel *Mytilus galloprovincialis* using Trizol (INVITROGEN) and the polyadenylated mRNA fraction was isolated by means of the MicroPoly(A) Purist system (AMBION). The cDNAs encoding H2A.X and H2A.Z genes (MgH2A.X and MgH2A.Z, respectively) were amplified through RT-PCR experiments using the SuperScript First-Strand Synthesis System (INVITROGEN), and the rapid amplification of 3′ and 5′ cDNA ends (RACE) was performed by using the SMART RACE cDNA amplification Kit (CLONTECH). In order to obtain the complete cDNA sequences of *Mytilus* MgH2A.X and MgH2A.Z genes, specific primers were designed using molecular databases from oysters [44] and mussels [45], detailed in Table 1. Agarose gel-purified PCR products were ligated into pCR2.1-TOPO vectors (INVITROGEN) and transformed into TOP10 competent cells (INVITROGEN). The plasmids were subsequently purified with the QIAprep Miniprep kit (QUIAGEN) and inserts were sequenced in a CEQ8000 sequencer (BECKMAN COULTER).

**Table 1:** Primers used in RT-PCR and RACE amplifications of H2A.X and H2A.Z genes (GSP, gene-specific primer; NGSP, nested gene-specific primer).

Histone	Primer	Sequence (5′-3′)
**H2A.X**	Fw-Oyst	CAGGTGCCCCTGTATATTTAGC
	Rv-Oyst	TCCTGGGACTGTGACGA
	GSP1	CGATCCTCCAGCAGCTTTCTTTGAC
	NGSP1	CACAGATCCCAATAGTCTATTAAG
	GSP2	TATCTTGCAGCAGAAGTGCTTG
	NGSP2	TCCAAGACATATACAACTAGCCATC
**H2A.Z**	GSP1	GTCTTCTGTGATCCCTTTTTGC
	NGSP1	ATGTGGAATGACACCACCACCA
	GSP2	GGCGGTAAAGCGGGAA
	NGSP2	AAGCTGTTTCTCGTTCCCAGA

### Inference of the phylogenetic relationships among H2A variants

All molecular and evolutionary analyses in this work were conducted using the program MEGA ver. 4.0 [46]. The phylogeny of H2A proteins was reconstructed from the obtained *p*-distances using the neighbor-joining method [47]. Phylogenetic inference analyses were completed by the reconstruction of maximum-parsimony trees [48] using the close-neighbor-interchange (CNI) search method. The reliability of the tree was tested by bootstrap [49] and the interior branch-test [50] methods, producing the bootstrap probability (BS) and the confidence probability (CP) values for each internal branch. Histone H2A sequences of the diplomonad protist Giardia were used as outgroups, given that this lineage is believed to be the first to diverge from all other eukaryotes [51].

### Protein expression and purification of recombinant H2A.X and H2A.Z histone variants

The coding regions of MgH2A.X and MgH2A.Z were independently cloned into pET11 expression vectors (NOVAGEN) and subsequently introduced into Rosetta (DE3) *Escherichia coli* (NOVAGEN) cells. The bacteria were grown in 0.5 L LB medium to an A600 of 0.8, adding isopropyl-beta-D-thiogalactoside to a final concentration of 1 mM. Cells were harvested after 14 h by centrifugation at 5000 g for 10 min at 4°C, and cell pellets were resuspended in 6 M GuHCl, 1 mM EDTA, 1 mM DTT, 50 mM Tris-HCl (pH 7.5) buffer and homogenized with a dounce. The cell lysate was dialyzed against 2 L of 0.1 M NaCl, 50 mM Tris-HCl (pH 7.5), 1 mM EDTA buffer for 2 h at 4°C. After dialysis, HCl was added to a final concentration of 0.5 N and cells were centrifuged at 11000 g for 15 min at 4°C. Six volumes of acetone were added to the supernatant and proteins were precipitated overnight at −20°C. The next day, the sample was centrifuged at 12000 g for 10 min at 4°C, dried under vacuum and resuspended in water. The HCl extracted histones were further purified using a Macro-Prep CM Cation Exchange resin (BIORAD) with a 0 to 1 M NaCl gradient in 7 M urea, 20 mM NaOAc (pH 5.2), 5 mM beta-mercaptoethanol, 1 mM EDTA buffer. The eluted MgH2A.X and MgH2A.Z proteins were dialyzed, lyophilized and further purified by reversed-phase HPLC using a Vydac C18 as described elsewhere [52].

### Isolation of native histones from *Mytilus*

Protein extractions and purifications were performed as described elsewhere [53]. Briefly, male gonad and hepatopancreas from *Mytilus* were independently homogenized with a dounce homogenizer in 150 mM NaCl, 10 mM Tris-HCl (pH 7.5), 0.5% Triton X-100 buffer containing a protease inhibitor mixture. After homogenization the samples were centrifuged at 3500 g in Corex tubes. The pellets were resuspended in 150 mM NaCl, 10 mM Tris-HCl (pH 7.5) and centrifuged again at 3500 g. The resulting pellets were resuspended in 0.6 N HCl, homogenized as above and centrifuged in the same way. The HCl supernatant extracts were precipitated with 6 volumes of acetone at −20°C overnight and then centrifuged at 12000 g for 10 min at 4°C. The acetone pellets were dried using a speedvac concentrator and stored at −80°C until further use. In order to separate histone H1 and Protamine-Like (PL) proteins from core histones, further fractionation of the acid soluble fraction was performed by using chromatography techniques and extraction with percloric acid [54]. The protein extract obtained (eluted core histones) was further purified by reversed-phase HPLC using a Vydac C18 [52].

### Polyacrylamide Gel Electrophoresis (PAGE)

Proteins were analyzed by AUT-PAGE gels (10% acrylamide, 0.5% bis-acrylamide, 5% acetic acid, 5.25 M urea, 5 mM Triton X-100) as described previously [55], and by SDS-PAGE gels (15% acrylamide, 0.4% bis-acrylamide) as described elsewhere [56]. Gels were stained with 0.2% (w/v) Coomassie blue in 25% (v/v) 2-propanol, 10% (v/v) acetic acid, and destained in 10% (v/v) 2-propanol, 10% (v/v) acetic acid. Nucleosome core particles were analyzed by 4% native PAGE gels (29∶1 acrylamide∶bisacrylamide) in 20 mM sodium acetate, 1 mM EDTA, 40 mM Tris-HCl (pH 7.2) buffer as described elsewhere [57], [58]. Native PAGE gels were stained with ethidium bromide and visualized with UV light. Two-dimensional gel electrophoresis analysis were carried out as described previously [17]. Briefly, histones were electrophoresed on a 10% polyacrylamide AUT gel in several lanes, one of which was cut out and soaked in 125 mM Tris-HCl (pH 8.8), 4% SDS, 20% glycerol, 1.43 M beta-mercaptoethanol buffer while the other was stained with Coomassie blue solution. The unstained gel strip was laid horizontally and electrophoresed in a 6% polyacrylamide stacking, 15% polyacrylamide separating SDS gel.

### H2A.X/H2A.Z-specific antibodies and Western blot analyses

The detection of H2A variants in different tissues from the mussel *Mytilus* was initially carried out by using human commercial anti-H2A.X (ABM) and anti-H2A.Z antibodies (ABCAM). In addition, *Mytilus*-specific anti-H2A.X and anti-H2A.Z antibodies were raised against polypeptide segments from these proteins displaying high antigenicity indexes after the application of the Kolaskar and Tongaonkar antigenicity scale [59]. The polypeptides chosen for antibody production were defined as SQSQEF (in the case of H2A.X) and KAKAVSR (for H2A.Z), located at protein regions specific for these variants and different from canonical H2A proteins. The polypeptides were synthesized at the Peptide Services of the University of Calgary (Canada) and the antibodies were raised in the animal care facility of the University of Victoria (Canada). In the present work, Western blot analyses were performed in order to detect the presence of MgH2A.X and MgH2A.Z proteins *in vivo*. To this end, native histones were separated in 10% SDS-PAGE gels, electro-transferred to a polyvinylidene difluoride (PVDF) membrane (BIORAD), and processed as described elsewhere [60]. Both commercial (unspecific) and *Mytilus*-specific anti-H2A.X and anti-H2A.Z antibodies were used at a 1∶3000 dilution, membranes were incubated with secondary goat anti-rabbit antibodies (GE HEALTHCARE) at a 1∶5000 dilution, and secondary antibodies were detected with enhanced chemiluminescence (GE HEALTHCARE) and exposure to X-ray films. The obtained blots were further probed with an in-house made antibody (Dr. Ausio's lab) raised against *Mytilus* histone H4, used as a loading control at a 1∶10000 dilution.

### Nucleosome core particle reconstitutions

Three types of *Mytilus*-specific nucleosome configurations were reconstituted in the present work by mixing native canonical H2B, H3 and H4 histones with either (a) native canonical H2A from *Mytilus*, (b) recombinant MgH2A.X, and (c) recombinant MgH2A.Z. Three rounds of histone protein titrations were carried out using SDS-PAGE to ensure that all histones were present in equimolar amounts in the final mixtures. Each mixture thus obtained was lyophilized and then resuspended in 6 M guanidinium-hydrochloride, 20 mM mercaptoethanol, 50 mM Tris-HCl (pH 7.5) buffer (1 mg/ml). After 30 min incubation at room temperature, each sample was dialyzed against 2 L of distilled water for 4 h, and then against 2 M NaCl, 50 mM Tris-HCl (pH 7.5), 1 mM EDTA, 1 mM DTT buffer overnight at 4°C [52]. After renaturation, histone octamers were reconstituted onto a 193 bp DNA fragment belonging to the promoter of the hsp90-1 gene from the mussel *M. galloprovincialis* [34]. Nucleosome positioning predictions for this DNA sequence were carried out by using the NuPoP software [61]. The DNA fragment was prepared by digesting the plasmid containing the 193 bp fragment with EcoRI followed by gel filtration chromatography, and DNA purification was conducted by repeated phenol/chloroform extractions and ethanol precipitation. Histones and DNA were then mixed at a histone∶DNA ratio of 1.13∶1 (w/w) in 2 M NaCl buffer. Nucleosome core particle reconstitutions were carried out using a stepwise salt gradient dialysis (2 to 0 M) in 20 mM Tris-HCl (pH 7.5), 0.1 mM EDTA (pH 8.0) buffer as described elsewhere [62]. The integrity of the core particles reconstituted was analyzed in 4% native PAGE gels.
